# Impact of the COVID-19 Pandemic on Administration of Selected Routine Childhood and Adolescent Vaccinations — 10 U.S. Jurisdictions, March–September 2020

**DOI:** 10.15585/mmwr.mm7023a2

**Published:** 2021-06-11

**Authors:** Bhavini Patel Murthy, Elizabeth Zell, Karen Kirtland, Nkenge Jones-Jack, LaTreace Harris, Carrie Sprague, Jessica Schultz, Quan Le, Cristi A. Bramer, Sydney Kuramoto, Iris Cheng, Mary Woinarowicz, Steve Robison, Ashley McHugh, Stephanie Schauer, Lynn Gibbs-Scharf

**Affiliations:** ^1^Immunization Services Division, National Center for Immunization and Respiratory Diseases, CDC; ^2^Stat-Epi Associates, Inc., Ponte Vedra Beach, Florida; ^3^Peraton Corporation, Herndon, Virginia; ^4^Idaho Department of Health and Welfare; ^5^Iowa Department of Public Health; ^6^Louisiana Department of Health; ^7^Michigan Department of Health and Human Services; ^8^Minnesota Department of Health; ^9^New York City Department of Health and Mental Hygiene, New York; ^10^North Dakota Department of Health; ^11^Oregon Health Authority; ^12^Washington State Department of Health; ^13^Wisconsin Department of Health Services.

After the March 2020 declaration of the COVID-19 pandemic in the United States, an analysis of provider ordering data from the federally funded Vaccines for Children program found a substantial decrease in routine pediatric vaccine ordering (*1*), and data from New York City and Michigan indicated sharp declines in routine childhood vaccine administration in these areas (*2*,*3*). In November 2020, CDC interim guidance stated that routine vaccination of children and adolescents should remain an essential preventive service during the COVID-19 pandemic (*4*,*5*). To further understand the impact of the pandemic on routine childhood and adolescent vaccination, vaccine administration data during March–September 2020 from 10 U.S. jurisdictions with high-performing* immunization information systems were assessed. Fewer administered doses of routine childhood and adolescent vaccines were recorded in all 10 jurisdictions during March–September 2020 compared with those recorded during the same period in 2018 and 2019. The number of vaccine doses administered substantially declined during March–May 2020, when many jurisdictions enacted stay-at-home orders. After many jurisdictions lifted these orders, the number of vaccine doses administered during June–September 2020 approached prepandemic baseline levels, but did not increase to the level that would have been necessary to catch up children who did not receive routine vaccinations on time. This lag in catch-up vaccination might pose a serious public health threat that would result in vaccine-preventable disease outbreaks, especially in schools that have reopened for in-person learning. During the past few decades, the United States has achieved a substantial reduction in the prevalence of vaccine-preventable diseases driven in large part to the ongoing administration of routinely recommended pediatric vaccines. These efforts need to continue even during the COVID-19 pandemic to reduce the morbidity and mortality from vaccine-preventable diseases. Health care providers should assess the vaccination status of all pediatric patients, including adolescents, and contact those who are behind schedule to ensure that all children are fully vaccinated.

Immunization information systems are confidential, computerized, population-based databases that consist of consolidated data on provider-administered vaccinations collected from 64 jurisdictions^†^ nationwide. Information from these systems can be used to track administered vaccines and measure vaccination coverage (*6*). Data were analyzed from 10 jurisdictions (Idaho, Iowa, Louisiana, Michigan, Minnesota, New York City, North Dakota, Oregon, Washington, and Wisconsin) with high-performing immunization information systems.

Numbers of vaccine doses administered weekly were measured during two periods: March–May 2020, and June–September 2020. These two periods were selected because many jurisdictions implemented and then lifted stay-at-home orders during these periods. During March–May 2020, eight of the 10 jurisdictions implemented some form of stay-at-home order (no orders were issued in Iowa and North Dakota) (Supplementary Table, https://stacks.cdc.gov/view/cdc/106855) (*7*). For each jurisdiction, the weekly percent change between the number of vaccine doses administered in 2020 and those administered in 2018 and 2019 was calculated. In addition, for each jurisdiction, the median and range of the weekly percent change for March–May 2020 and June–September 2020 were calculated. Finally, the overall median of the median weekly percentage was calculated for each period (referred to as the median in this report) to determine the overall impact across all 10 jurisdictions. The following routinely recommended childhood and adolescent vaccines,**^§^** by targeted age groups, were analyzed: diphtheria, tetanus, and acellular pertussis (DTaP) for children aged 0–23 months and children aged 2–6 years; measles, mumps, and rubella (MMR) for children aged 12–23 months and children aged 2–8 years; human papillomavirus (HPV) for children aged 9–12 years and adolescents aged 13–17 years; and tetanus, diphtheria and acellular pertussis (Tdap) for adolescents aged 13–17 years. This activity was reviewed by CDC and was conducted consistent with applicable federal law and CDC policy.^¶^

During March–May 2020, vaccine doses administered to children and adolescents substantially decreased for all vaccines examined across the 10 jurisdictions compared with the same period in 2018 and 2019 (Table 1) (Table 2). Among children aged <24 months and children aged 2–6 years, DTaP doses administered declined an overall median of 15.7% and 60.3%, respectively, across all jurisdictions compared with the same period during 2018 and 2019 (Table 1). During March–May 2020, MMR doses administered to children aged 12–23 months and children aged 2–8 years declined a median of 22.4% and 63.1%, respectively. Among children aged 9–12 years and adolescents aged 13–17 years, HPV doses administered declined a median of 63.6% and 71.3%, respectively during March–May 2020 compared with doses administered during the same period in 2018 and 2019 (Table 2). Doses of Tdap administered during this period in 2020 decreased a median of 66.4% among children aged 9–12 years and 61.4% among adolescents aged 13–17 years compared with 2018 and 2019.

**TABLE 1:** Median weekly percent change* in diphtheria, tetanus, and acellular pertussis vaccine doses administered to children aged <24 months and aged 2–6 years and in measles, mumps, and rubella vaccine doses administered to children aged 12–23 months and aged 2–8 years compared with the average number of doses administered during the same period in 2018 and 2019 — 10 U.S. jurisdictions, March–September 2020

Vaccine/Age/U.S. jurisdiction	% Change, median (range)			
	Mar–May		Jun–Sep	
**DTaP vaccine**				
Age group	<24 mos	2–6 yrs	<24 mos	2–6 yrs
Idaho	−8.7 (−32.1 to 7.0)	−39.2 (−75.2 to −11.2])	−11.0 (−23.2 to 4.8)	−4.3 (−28.3 to 42.7)
Iowa	−15.7 (−35.7 to 1.1)	−51.5 (−75.3 to 0.6)	−10.4 (−32.5 to 9.7)	−10.5 (−31.4 to 33.9)
Louisiana	−11.6 (−46.3 to 9.9)	−57.9 (−85.3 to 7.2)	−9.0 (−36.6 to 14.5)	−4.3 (−39.2 to 29.5)
Michigan	−21.6 (−55.6 to −6.1)	−62.6 (−88.2– to −7.8)	−6.9 (−24.4 to 15.7)	−10.7 (−33.6 to 26.5)
Minnesota	−15.7 (−41.2 to −6.7)	−63.4 (−91.0 to 7.0)	−10.5 (−33.6 to 16.7)	−7.0 (−28.2 to 25.9)
New York City	−27.2 (−66.1 to −3.6)	−74.9 (−94.5 to −4.1)	−7.5 (−26.8 to 4.1)	−6.4 (−37.2 to 16.1)
North Dakota	−11.1 (−41.3 to 0.0)	−20.5 (−76.7 to 23.6)	−6.5 (−18.6 to 19.6)	24.9 (−6.9 to 106.3)
Oregon	−14.1 (−38.3 to −3.9)	−60.6 (−81.1 to −12.3)	−9.2 (−41.5 to 10.4)	−8.4 (−51.4 to 18.9)
Washington	−17.0 (−42.1 to −5.0)	−60.1 (−82.9 to −20.0)	−8.5 (−30.4 to −0.6)	−9.0 (−28.0 to 6.5)
Wisconsin	−21.6 (−38.8 to 9.2)	−70.8 (−90.3 to −7.2)	−9.5 (−22.8 to 2.1)	−5.3 (−27.4 to 27.3)
**Median of medians** **^†^**	**−15.7**	**−60.3**	**−9.1**	**−6.7**
**MMR vaccine**				
Age group	12–23 mos.	2–8 yrs	12–23 mos	2–8 yrs
Idaho	−15.3 (−48.8 to12.4)	−39.8 (−77.5 to −11.3)	−8.8 (−20.7 to 11.5)	−7.1 (−30.4 to 42.5)
Iowa	−23.7 (−38.8 to −4.7)	−52.4 (−74.9 to 0.7)	−8.8 (−30.5 to 17.2)	−11.6 (−32.1 to 34.3)
Louisiana	−18.0 (−53.5 to 4.3)	−59.9 (−85.1 to 4.5)	−12.1 (−47.6 to 12.0)	−5.1 (−41.4 to 27.2)
Michigan	−32.3 (−66.4 to −6.9)	−65.3 (−90.9 to −8.9)	−6.8 (−25.3 to 29.0)	−16.9 (−34.6 to 20.8)
Minnesota	−22.5 (−51.5 to −9.5)	−66.5 (−92.3 to −2.9)	−9.0 (−31.4 to 9.1)	−3.4 (−26.8 to 33.1)
New York City	−42.8 (−80.6 to −10.4)	−82.2 (−95.5 to −17.4)	−7.1 (−25.9 to 8.0)	−20.5 (−52.2 to 11.5)
North Dakota	−22.3 (−47.0 to 21.0)	−55.3 (−80.4 to 6.0)	−7.2 (−18.5 to 35.5)	−11.0 (−22.0 to 46.0)
Oregon	−20.8 (−58.4 to 4.4)	−62.7 (−82.4 to −24.5)	−9.8 (−43.1 to 12.8)	−15.6 (−54.8 to 7.7)
Washington	−19.2 (−55.7 to −2.3)	−63.5 (−85.3 to −27.8)	−9.3 (−32.8 to −1.3)	−17.2 (−37.3 to −2.5)
Wisconsin	−25.2 (−54.7 to 19.2)	−74.4 (−91.1 to −11.0)	−5.2 (−28.3 to 23.5)	−8.0 (−28.3 to 22.5)
**Median of medians** **^†^**	**−22.4**	**−63.1**	**−8.8**	**−11.3**

**Abbreviations:** DTaP = diphtheria, tetanus, and acellular pertussis; MMR = measles, mumps, and rubella.

* For each jurisdiction, the weekly percentage change between the number of vaccine doses administered March–September 2020 and those administered March–September in 2018 and 2019 was calculated. In addition, for each jurisdiction the median and range of the weekly percent change for March–May 2020 and June–September 2020 were calculated.

† The overall median of the median weekly percentage was calculated for each period to determine the overall impact across all 10 jurisdictions.

**TABLE 2:** Median weekly percent change* in human papillomavirus vaccine doses administered to children aged 9–12 years and adolescents aged 13–17 years and change in tetanus, diphtheria, and acellular pertussis vaccine doses administered to children aged 9–12 years and adolescents aged 13–17 years compared with the average number of doses administered during the same period in 2018 and 2019 — 10 U.S. jurisdictions, March–September 2020

Vaccine/Age/U.S. jurisdiction	% Change, median (range)			
	Mar–May		Jun–Sep	
**HPV vaccine**				
Age group	9–12 yrs	13–17 yrs	9–12 yrs	13–17 yrs
Idaho	−25.8 (−63.8 to 14.3)	−38.5 (−71.5 to −3.3)	−3.8 (−34.5 to 94.3)	−8.1 (−28.6 to 92.7)
Iowa	−55.6 (−79.0 to 9.4)	−65.5 (−84.1 to −18.2)	−3.0 (−29.0 to 90.4)	−25.2 (−44.1 to 22.2)
Louisiana	−54.1 (−86.7 to 0.3)	−66.7 (−88.5 to −3.2)	−13.9 (−46.5 to 17.0)	−23.4 (−59.6 to −6.5)
Michigan	−69.7 (−89.2 to 1.5)	−76.5 (−92.4 to −13.7)	−14.7 (−30.7 to 48.1)	−36.9 (−44.5 to 13.2)
Minnesota	−66.1 (−88.5 to 21.2)	−77.7 (−92.4 to 6.9)	−14.0 (−38.4 to 134.5)	−32.4 (−58.8 to 86.5)
New York City	−82.5 (−95.5 to 8.3)	−85.5 (−96.0 to −5.1)	−8.4 (−50.2 to 13.8)	−25.1 (−63.8 to 2.2)
North Dakota	−44.2 (−87.3 to 26.3)	−57.4 (−87.6 to 7.8)	−1.9 (−26.8 to 84.3)	−20.7 (−48.0 to 70.7)
Oregon	−65.0 (−85.5 to 17.5)	−69.2 (−86.9 to −7.6)	−12.8 (−38.3 to 42.2)	−37.6 (−54.2 to 24.9)
Washington	−62.1 (−89.4 to −4.2)	−73.3 (−89.0 to −3.9)	−28.2 (−60.5 to −2.6)	−40.9 (−58.4 to −7.2)
Wisconsin	−75.2 (−92.2 to 6.8)	−81.7 (−94.2 to −10.5)	−11.6 (−30.8 to 78.0)	−30.9 (−52.1 to 64.4)
**Median of medians** **^†^**	**−63.6**	**−71.3**	**−12.2**	**−28.1**
**TdaP vaccine**				
Age group	9–12 yrs	13–17 yrs	9–12 yrs	13–17 yrs
Idaho	−36.9 (−67.7 to 3.3)	−31.5 (−59.6 to 13.3)	−13.8 (−43.2 to 101.3)	−4.7 (−33.3 to 60.7)
Iowa	−60.3 (−79.7 to 4.6)	−61.9 (−84.7 to −25.9)	−7.7 (−29.4 to 87.0)	−33.3 (−43.5 to 16.7)
Louisiana	−60.1 (−88.5 to −7.8)	−58.9 (−81.3 to 10.9)	−22.0 (−47.2 to 16.8)	−30.1 (−55.0 to 20.6)
Michigan	−68.1 (−89.8 to −2.7)	−63.0 (−79.6 to 8.9)	−21.9 (−38.8 to 39.8)	−39.6 (−50.1 to −14.7)
Minnesota	−72.3 (−89.8 to 2.5)	−74.4 (−82.5 to 6.2)	−27.8 (−46.3 to 113.8)	−29.8 (−50.9 to 13.2)
New York City	−83.9 (−96.4 to −4.6)	−91.2 (−95.3 to 1.0)	−18.2 (−51.2 to 0.3)	−54.6 (−73.9 to −40.3)
North Dakota	−44.1 (−85.9 to 22.8)	−22.2 (−58.8 to 92.3)	−5.8 (−25.8 to 106.1)	59.4 (−51.4 to 210.5)
Oregon	−66.9 (−87.5 to −1.9)	−60.8 (−79.1 to −8.3)	−22.5 (−49.2 to 24.7)	−41.8 (−59.0 to −19.4)
Washington	−65.9 (−88.8 to −17.8)	−65.5 (−78.0to 27.5)	−38.1 −70.8 to−20.7)	−26.7 (−51.7 to 5.2)
Wisconsin	−74.5 (−92.3 to 0.5)	−49.6 (−73.8 to 3.5)	−20.7 (−35.9 to 70.6)	−21.5 (−40.9 to 21.1)
**Median of medians** **^†^**	**−66.4**	**−61.4**	**−21.3**	**−30.0**

**Abbreviations**: HPV = human papillomavirus; TdaP = tetanus, diphtheria, and acellular pertussis.

* For each jurisdiction, the weekly percentage change between the number of vaccine doses administered March–September 2020 and those administered March–September in 2018 and 2019 was calculated. In addition, for each jurisdiction the median and range of the weekly percent change for March–May 2020 and June–September 2020 were calculated.

† The overall median of the median weekly percentage was calculated for each period to determine the overall impact across all 10 jurisdictions.

During June–September 2020, after most stay-at-home orders had been lifted, the number of weekly routine pediatric vaccine doses administered increased initially, approaching or even surpassing baseline prepandemic levels in most of the 10 jurisdictions, with some differences by jurisdiction, vaccine type, and age. However, across all age groups and across all vaccine types, none of the jurisdictions demonstrated a sustained or prolonged increase in the number of weekly doses administered above prepandemic administration levels, which would have been necessary to catch up children and adolescents who missed routine vaccinations (Figure). Among children aged <24 months and aged 2–6 years, administration of DTaP vaccine declined a median of 9.1% and 6.7%, respectively during June–September 2020, compared with the same period during 2018 and 2019. Among children aged 12–23 months and aged 2–8 years, administration of MMR vaccine decreased 8.8% and 11.3%, respectively compared with 2018 and 2019. During the same period, among children aged 9–12 years and adolescents aged 13–17 years, administration of HPV vaccine decreased a median of 12.2% and 28.1%, respectively, and among the same age groups, Tdap vaccine administration decreased a median of 21.3% and 30.0%, respectively, compared with the average during the same period in 2018 and 2019.

**FIGURE:**
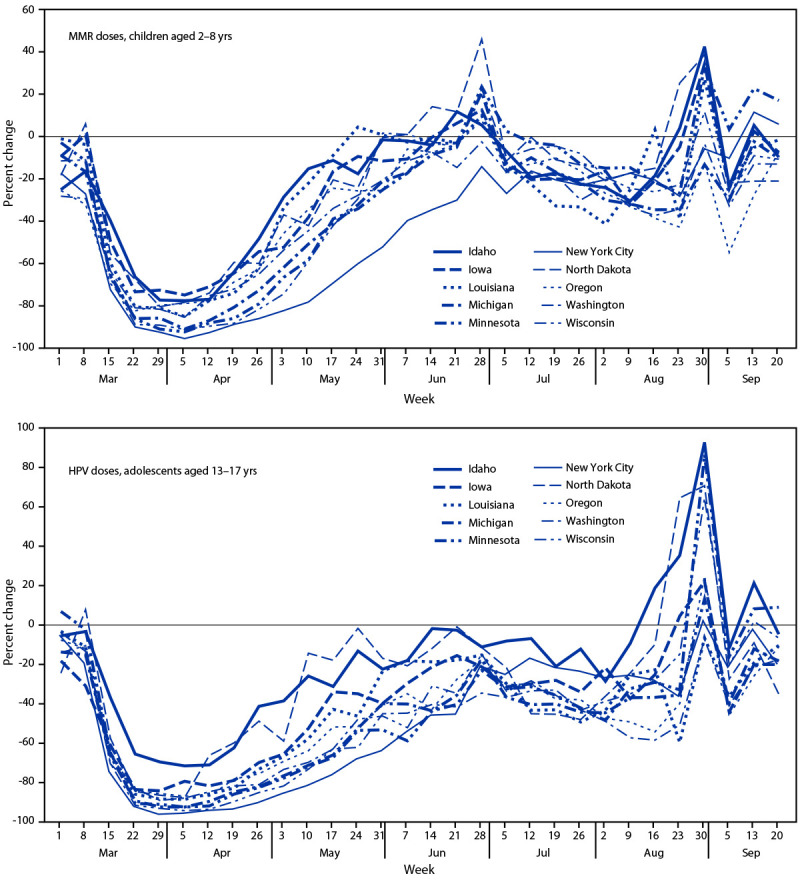
Percent change in measles, mumps, and rubella vaccine doses administered to children aged 2–8 years and in human papillomavirus vaccine doses administered to adolescents aged 13–17 years compared with the average number of doses administered during the same period in 2018 and 2019 — 10 U.S. jurisdictions,* March–September 2020 **Abbreviations**: HPV = human papillomavirus; MMR = measles, mumps, and rubella. * During March–May 2020, eight of the 10 jurisdictions implemented some form of stay-at-home order; no orders were issued in Iowa and North Dakota.

## Discussion

In 10 U.S. jurisdictions with high-performing immunization information systems, vaccine administration data indicated that administered doses of routine childhood and adolescent vaccines were substantially lower during March–May 2020 compared with the average administered during the same period in 2018 and 2019. This decline, which is consistent with other data sources indicating a similar decrease in routine pediatric vaccine ordering, corresponded to the enactment of stay-at-home orders in many jurisdictions (*1*). Although vaccine administration rebounded during June–September 2020, approaching prepandemic levels in most jurisdictions, this increase was not sufficient to achieve the catch-up vaccination needed to address the many months when children missed routine vaccination.

Several factors might explain this lag in catch-up vaccination. Depending on case rates in certain jurisdictions, fear of contracting COVID-19 in the health care facility or the community during the pandemic might have prevented some parents from seeking routine pediatric care for their children (*8*). Jurisdictions might have differed in the duration or enforcement of stay-at-home orders or in the prevalence of COVID-19 cases at different time points. In addition, the rapid transition to virtual learning because of the COVID-19 pandemic might have resulted in a lack of enforcement of immunization requirements for school attendance.

Routine child and adolescent vaccination remains an important cornerstone of public health practice and is a critical frontline tool in the prevention of morbidity and mortality in younger populations. Even a transient decline in vaccination coverage can compromise herd immunity and result in the propagation of outbreaks. During 2018–2019, a measles outbreak occurred in Rockland County, New York and nearby counties. Measles vaccination coverage in schools in the affected area was only 77%, far below the 93%–95% coverage needed to sustain measles herd immunity (*9*,*10*). Pediatric outbreaks of vaccine-preventable diseases have the potential to derail efforts to reopen schools for the 2021–22 academic year and further delay nationwide efforts to return students to the classroom. Health care systems and other social institutions are already overburdened by the COVID-19 pandemic, and vaccine preventable disease outbreaks can lead to loss of in-person learning and further overwhelm community resources and contribute to morbidity and mortality. As COVID-19 vaccinations become readily available to pediatric populations, CDC recommends providers consider co-administering COVID-19 vaccines with other routinely recommended vaccines, especially when patients are behind or might fall behind on routine recommended vaccines.^**^

The findings in this report are subject to at least three limitations. First, vaccination data from only 10 U.S. jurisdictions were analyzed and, therefore, these findings might not be generalizable to the entire United States. Second, immunization information systems were the only type of system analyzed, and vaccination information was not corroborated with other surveillance programs, such as National Immunization Survey-Child^††^ or National Immunization Survey-Teen.^§§^ Finally, the specific reasons for the decrease in vaccine administration during March–May 2020, or the rebound in vaccine administration during June–September 2020 in any of the 10 jurisdictions could not be determined. Vaccine administration could have increased because stay-at-home orders were lifted, the jurisdiction actively conducted catch-up campaigns, or the jurisdiction faced issues with vaccine storage and handling, which would have required revaccination of those patients who received invalid doses, among other reasons.

To facilitate the safe reopening of schools for in-person learning, CDC issued a call to action^¶¶^ in early 2021 encouraging health care systems, health care providers, schools, parents, and state and local governments to work together to ensure that students are caught up on all routinely recommended vaccinations. High vaccination coverage rates help protect pediatric populations and ensure that herd immunity is maintained for all vaccine-preventable diseases. The COVID-19 pandemic has substantially disrupted routine medical care in the United States, requiring a consolidated and coordinated effort among multiple partners to promote catching up and staying up to date on routine vaccinations for children of all ages. Health care providers should assess the vaccination status of all pediatric patients, including adolescents, and contact those who are behind schedule to ensure that all children are fully vaccinated.

**Summary**

**What is already known about this topic?**

Early reports during the COVID-19 pandemic documented a marked decline in pediatric vaccine ordering and administration, placing U.S. children and adolescents at risk for vaccine-preventable diseases.

**What is added by this report?**

Analysis of immunization information systems data from 10 U.S. jurisdictions indicated a substantial decrease in administered vaccine doses during March–May 2020 compared with the same period during 2018 and 2019. Although administered doses increased during June–September 2020, this increase was not sufficient to achieve catch-up coverage.

**What are the implications for public health practice?**

To prevent outbreaks of vaccine-preventable diseases, health care providers should assess the vaccination status of all pediatric patients, including adolescents, and contact those who are behind schedule to ensure that all children and adolescents are fully vaccinated.

## Footnotes

* A high-performing immunization information system was defined as a system with vaccine estimates within 10 percentage points of those from the 2018 National Immunization Survey-Child and National Immunization Survey-Teen immunization information systems, and which recorded ≥90% of doses administered to persons aged <19 years that were submitted and processed within 30 days of vaccine administration.

† The 64 immunization information systems jurisdictions consist of 50 U.S. states, eight U.S. territories and freely associated states (Puerto Rico, U.S. Virgin Islands, American Samoa, Northern Mariana Islands, Guam, Marshall Islands, Palau, and the Federated States of Micronesia), and six local jurisdictions (Chicago, Illinois; Houston, Texas; San Antonio, Texas; Philadelphia, Pennsylvania; New York City, New York; and Washington, DC).

§
https://www.cdc.gov/vaccines/schedules/hcp/imz/child-adolescent.html

¶ 45 C.F.R. part 46.102(l)(2), 21 C.F.R. part 56; 42 U.S.C. Sect. 241(d); 5 U.S.C. Sect. 552a; 44 U.S.C. Sect. 3501 et seq.

**
https://www.cdc.gov/vaccines/covid-19/info-by-product/clinical-considerations.html

††
https://www.cdc.gov/vaccines/imz-managers/coverage/childvaxview/index.html

§§
https://www.cdc.gov/vaccines/imz-managers/coverage/teenvaxview/index.html

¶¶
https://www.cdc.gov/vaccines/partners/index.html
